# Glottal function index as a brief screening tool: Determination of an optimal cutoff score for functional dysphonia

**DOI:** 10.1007/s00405-026-10167-2

**Published:** 2026-03-28

**Authors:** Cigdem Onen, Hakan Golac, Guzide Atalik, Adnan Gulacti, Fatma Esen Aydinli, Metin Yilmaz

**Affiliations:** 1 Department of Speech and Language Therapy, Ankara Yildirim Beyazit University, Ankara, Türkiye; 2 Department of Speech and Language Therapy, Gazi University, Ankara, Türkiye; 3 Department of Speech and Language Therapy, Hacettepe University, Ankara, Türkiye; 4 Department of Otorhinolaryngology and Head-Neck Surgery, Gazi University, Ankara, Türkiye; 5 Ankara Yildirim Beyazit University Faculty of Health Sciences, Department of Speech and Language Therapy, Esenboga, Ankara, Türkiye; 6 Gazi University Faculty of Health Sciences, Department of Speech and Language Therapy, Cankaya, Ankara, Türkiye; 7 Hacettepe University Faculty of Health Sciences, Department of Speech and Language Therapy, Sihhiye Ankara, Türkiye; 8 Gazi University Faculty of Medicine, Department of Otorhinolaryngology and Head-Neck Surgery, Besevler Ankara, Türkiye

**Keywords:** Functional dysphonia, Glottal Function Index, Self-reported measurements, Voice diagnostics, Voice screening

## Abstract

**Purpose:**

This study aimed to evaluate the diagnostic utility of the Glottal Function Index (GFI) in distinguishing individuals with functional dysphonia from normophonic controls and to determine an optimal cutoff score for screening.

**Methods:**

A total of 100 participants (50 males, 50 females) were included in this case–control study. The study group (SG) comprised 50 patients with functional dysphonia, and the control group (CG) included 50 age- and gender-matched healthy individuals. All participants underwent a multidimensional voice assessment, including acoustic analysis, auditory-perceptual evaluation (GRBAS), aerodynamic measurement [Maximum Phonation Time (MPT)], and self-reported outcome measures [Voice Handicap Index-10 (VHI-10), Voice-Related Quality of Life (V-RQoL), and GFI]. Group comparisons were performed, and receiver operating characteristic (ROC) analysis was used to determine the optimal GFI cutoff based on the Youden index.

**Results:**

ROC analysis demonstrated excellent diagnostic accuracy for the GFI in functional dysphonia (AUC = 0.983). The optimal cutoff score was ≥ 3, providing 96% sensitivity and 96% specificity. The SG exhibited significantly higher GFI scores [median 14 (9.75–19)] than the CG [0 (0–0)] (*p* < 0.001). The SG also showed significantly deteriorated acoustic (increased shimmer, decreased harmonics-to-noise ratio), auditory-perceptual (higher G score), aerodynamic (shorter MPT), and self-reported (higher VHI-10, lower V-RQoL) parameters than the CG (all *p* < 0.001).

**Conclusion:**

The GFI is a highly sensitive self-report tool for identifying functional dysphonia. A cutoff score of ≥ 3 provides excellent diagnostic accuracy and supports its use as a brief screening measure within multidimensional voice assessment protocols.

## Introduction

Voice disorders affect approximately 3–9% of the general population and can significantly impact quality of life by impairing communication and social participation [1, 2]. These disorders are generally classified as either organic or non-organic [3]. Organic voice disorders result from structural or neurological issues in the larynx, whereas non-organic voice disorders are characterized by impaired phonation without identifiable organic pathology [3, 4]. However, excessive or maladaptive laryngeal muscle tension can also occur in the presence of an underlying organic lesion, a phenomenon referred to as secondary muscle tension dysphonia (MTD), in which the nature or severity of the organic pathology cannot fully explain the degree of muscular tension [5]. Non-organic voice disorders include psychogenic and functional types, which differ in their causes, but both lack structural or neurological origins [3].

Available evidence suggests a substantially higher prevalence of functional voice disorder compared to organic voice disorder in clinic-based and occupational samples [4, 6, 7], highlighting its clinical significance and the need for effective diagnostic approaches. Functional voice disorder is described in the literature using synonymous terms such as hyper- or hypofunctional dysphonia, non-organic dysphonia, or MTD [8, 9]. Its etiology is multifactorial, often involving inappropriate phonatory technique, vocal misuse or overuse, excessive laryngeal tension, and stress [10–14].

Because voice disorders involve multiple physiological and perceptual dimensions, clinical assessment typically requires a multidimensional approach that integrates both objective and subjective measures. These include laryngeal imaging techniques (e.g., transnasal fiberoptic laryngoscopy, videostroboscopy), auditory-perceptual evaluation (e.g., the Grade, Roughness, Breathiness, Asthenia, and Strain - GRBAS scale [15], the Consensus Auditory-Perceptual Evaluation of Voice - CAPE-V scale [16]), acoustic and aerodynamic measures, and patient-reported outcome measures (e.g., Voice-Related Quality of Life - V-RQoL [17], Voice Outcome Survey [18], Voice Activity and Participation Profile [19], Voice Symptom Scale [20], and Voice Handicap Index - VHI [21]).

Acoustic and aerodynamic measurements provide insights into both laryngeal efficiency and function [22–24]. Auditory-perceptual analysis enables clinicians to quantify the functional impact of voice disorders on voice quality [25–28]. Patient-reported quality of life scales capture the subjective perception of voice problems. These scales not only reflect the personal burden of dysphonia but also play a key role in treatment adherence [17, 29, 30]. Although objective assessments strengthen diagnosis and help evaluate intervention outcomes, the patient’s subjective impression of their own voice disorder remains a central determinant of clinical success [31]. Easily administered and multidimensional self-report tools are crucial for guiding treatment planning, setting expectations, and managing clinical workload.

The Glottal Function Index (GFI), developed and validated by Bach et al. [31], was specifically designed to fulfill this need. It is a more focused, brief, and easily self-administered questionnaire that evaluates the presence and degree of symptoms of glottal dysfunction. The GFI consists of four items assessing: (1) increased phonatory effort during speaking, (2) throat discomfort or pain after voice use, (3) vocal fatigue or voice weakening with prolonged speaking, and (4) voice breaks or changes in voice quality. Each item is rated on a 6-point scale ranging from 0 (asymptomatic) to 5 (severe), yielding a total score between 0 and 20 [31]. The Turkish version of the GFI (GFI-TR) has recently demonstrated good validity and reliability in adult populations [32, 33]. Due to its brevity and ease of administration, the GFI is particularly suited for initial screening and routine clinical monitoring.

Previous studies have demonstrated the ability of the GFI to distinguish cases with glottal insufficiency, typically in heterogeneous groups with diverse dysphonia etiologies [31, 34–39]. However, research focusing specifically on functional voice disorders remains limited. Establishing an optimal cutoff score for this population may enhance the clinical utility of the GFI as a brief screening tool.

In light of these findings, the present study aimed to evaluate the diagnostic discriminative power of the GFI in identifying functional dysphonia and to determine its optimal cutoff score for screening purposes.

## Materials and methods

### Study design and ethical considerations

This case–control study was conducted at the Prof. Dr. Necmettin Akyıldız Hearing, Speech, and Voice Center and approved by the research ethics committee of Gazi University (IRB number: 2025 − 1536). The research was explained to all participants, and written informed consent was obtained from each participant prior to enrollment.

### Participants

A total of 100 (50 males and 50 females) participants were included in the present study. The study group (SG) consisted of 50 patients (21 males and 29 females; mean ± SD age, 46.08 ± 13.19) who were diagnosed with functional voice disorders by an experienced Ear, Nose, and Throat (ENT) specialist. In the SG, a detailed videolaryngostroboscopy (VLS) was performed using a flexible rhinolaryngoscope (Olympus HD ENF-VH) connected to a video processor and light source system (Olympus EVIS EXERA III CV 190 video processor and Olympus CLL-S1 light source; Olympus Medical Systems Corp., Tokyo, Japan). The control group (CG) consisted of 50 age- and gender-matched healthy normophonic volunteers (21 males and 29 females; mean ± SD age, 46.08 ± 12.63). All included subjects were between 18 and 65 years of age. Participants were excluded if they had mucosal lesions, such as nodules, cysts, polyps, sulcus vocalis, or leukoplakia; laryngeal inflammatory disorders, including Reinke’s edema and chronic laryngitis; a neurogenic etiology of dysphonia (i.e., paresis, paralysis, or spasmodic dysphonia); or any vascular lesion of the vocal cords. Those with respiratory dysfunction, a history of laryngeal surgery or radiotherapy/chemotherapy to the head and neck, those receiving voice therapy, and those with symptoms of upper airway infection, allergies, or laryngopharyngeal reflux at the time of evaluation were also excluded from the study. Additionally, professional voice users were excluded since the questionnaires used in the present study are not specifically designed to measure vocal changes in this group.

All participants underwent a detailed voice assessment protocol, which included acoustic voice analysis, auditory-perceptual assessment, aerodynamic measurements, and self-reported outcomes.

### Voice recordings

The voice samples of all participants were collected in a sound-treated room with an ambient noise level of less than 40 dB using the Computerized Speech Lab (CSL) software (Model 3700, Version 3.4.1, 2000–2001 Kay PENTAX, Montvale, NJ) [40]. A Rode NT1 Cardioid Condenser microphone with a frequency range of 20 Hz to 20 kHz was used. The mouth-to-microphone distance was adjusted to 10 cm, and the microphone was positioned at a 90° angle to the mouth. Participants were positioned in a standing posture with hips and shoulders symmetrical and comfortable [41]. They were then asked to produce sustained phonation of the vowel /a/ three times for a minimum of 4 s, at their most comfortable pitch and loudness. Each participant was given a short practice period before the first recording to adapt to the procedure. All voice samples were recorded at a sampling frequency of 44,100 Hz and 16-bit resolution [41] and then saved as *“.wav”* format on a desktop computer.

### Acoustic analyses

Segments lasting at least 0.5 s from the beginning and end of the recorded voice samples were extracted using Adobe Audition CC (version 11.1) software to avoid unintended irregularities and variability at voice onset and offset [42]. Then, the most stable 3 s mid-portion of sustained phonation, representing the steady-state segment, was selected for the acoustic analysis, excluding onset and offset segments. The same selection criteria were applied consistently across all participants.

In our clinic, only the main CSL recording module is available; therefore, CSL-based acoustic analysis modules were not used. Acoustic analyses were subsequently performed using the Praat software [43], which has been widely validated and extensively used for acoustic voice analysis in clinical and research settings. The acoustic parameters, including mean fundamental frequency (F0), percent jitter, percent shimmer, and mean harmonics-to-noise ratio (HNR), were analyzed through the sustained phonation of the vowel /a/ using a voice quality measurement script, which was added to the Praat software (version 6.3.20) to obtain the objective voice quality measurements automatically [43–45]. Calibrated sound pressure level (SPL) measurements were not obtained in the present study. Relative vocal intensity as recorded by the CSL system was therefore used.

### Auditory-perceptual analyses

The GRBAS scale, developed by the Japanese Society of Logopedics and Phoniatrics, was used for the auditory-perceptual analysis. This scale contains five parameters of voice production: Grade (the overall voice quality), Roughness (irregular fluctuations of frequency), Breathiness (the turbulence caused by air leakage), Asthenia (voice weakness), and Strain (perception of excessive vocal effort). Each parameter is rated on a 4-point Likert scale by assigning a number ranging between 0 and 3 (normal, mild, moderate, and severe) [15]. In this study, only the G parameter of the GRBAS scale was used for auditory-perceptual ratings. Judgments were conducted by a Speech Language Therapist (SLT) with 10 years of experience in the assessment and treatment of voice disorders.

### Aerodynamic measurement

The Maximum Phonation Time (MPT) was obtained from all participants as an aerodynamic measurement. For MPT, participants were instructed to take a deep breath in a seated position and sustain the vowel /a/ for as long as possible at their most comfortable pitch and loudness. Three trials were performed by each participant, with a one-minute rest period between trials, and the longest phonation duration was recorded as the MPT. Prior to the measurements, the procedure was demonstrated by a clinician to ensure that participants clearly understood the task [46].

### Self-reported outcomes

All participants were asked to complete the VHI-10 [47], V-RQoL [48], and GFI [32, 33] questionnaires. The VHI-10 consists of 10 items and provides a multidimensional evaluation addressing the functional, emotional, and physical aspects of voice disorders. Each item is scored on a 5-point scale ranging from 0 to 4, yielding a total score between 0 and 40. Higher scores indicate greater perceived handicap, and a score of 7 or higher is considered indicative of a voice disorder [47]. The V-RQoL questionnaire consists of 10 items scored on a 5-point scale related to physical, functional, and socio-emotional aspects, and a higher total score indicates a better voice-related quality of life. The GFI is a brief screening tool for detecting voice disorders [32, 33]. The GFI results in a total score ranging from 0 to 20. A score of 4 or higher is considered suggestive of a voice disorder [31].

### Statistical analyses

Data were analyzed using the Statistical Package for the Social Sciences (SPSS) software, version 25.0 (SPSS Inc., Chicago, USA). The normality of the data distribution was assessed using both visual (histogram and probability graphs) and analytical methods (Kolmogorov-Smirnov/Shapiro-Wilk tests). Since all voice-related data, including acoustic, auditory-perceptual, aerodynamic, and self-reported outcomes, were not normally distributed, the Mann-Whitney *U* test was performed for all comparisons between the SG and CG. The discriminatory performance of the GFI questionnaire in differentiating normophonic participants from individuals with functional voice disorders was assessed using receiver-operating characteristic (ROC) analysis. The optimal cutoff value for GFI was identified based on the maximum Youden index (sensitivity + specificity − 1) and the maximized area under the curve (AUC) [49]. All statistical tests were two-tailed, and a p-value of < 0.05 was considered statistically significant for the present study.

## Results

The median (IQR) values of acoustic parameters differed significantly between the study and control groups, except for the mean F0 and percent jitter parameters. Percent shimmer was significantly higher in the SG compared to the CG (*p* < 0.001), while significantly higher mean HNR was obtained in the CG than in the SG (*p* < 0.001). Significantly higher G score of GRBAS and shorter MPT were found in the SG than in the CG, with *p* values < 0.001. The median (IQR) value of VHI-10 was 19 (7.5–29) in the SG and 0 (0–0) in the CG (*p* < 0.001). The median (IQR) value of the V-RQoL total score was significantly lower in the SG compared to the controls [46.25 (31.87–75.62) vs. 100 (100–100); *p* < 0.001]. A similar significant difference was also observed in the median (IQR) value of GFI scores between the groups, which was higher in the SG [14 (9.75–19) vs. 0 (0–0), *p* < 0.001]. Comparisons of all voice-related data between the groups are given in Table 1.

**Table 1:** Voice-related outcomes in the study and control groups

Parameters	Study group (*N* = 50) Median (IQR)	Control group (*N* = 50) Median (IQR)	*P* value
**Acoustic parameters** Mean F0 (Hz) Percent jitter (%) Percent shimmer (%) Mean HNR (dB)	197.62 (149.36–249.19) 0.26 (0.17–0.46) 4.69 (2.61–6.95) 21.15 (17.46–24.49)	208.73 (139.04–242.37) 0.21 (0.15–0.32) 2.26 (1.50–3.17) 24.84 (23.02–26.90)	0.798 0.133 < 0.001 < 0.001
**Auditory-perceptual ratings** Grade (G) of GRBAS	1 (1–2)	0 (0–0)	< 0.001
**Aerodynamic measurement** MPT (s)	10.07 (6.80–13.82)	18.63 (14.12–21.49)	< 0.001
**Self-reported outcomes** VHI-10 V-RQoL GFI	19 (7.5–29) 46.25 (31.87–75.62) 14 (9.75–19)	0 (0–0) 100 (100–100) 0 (0–0)	< 0.001 < 0.001 < 0.001

*Abbreviations*: *F0* fundamental frequency, *HNR* harmonic-to-noise ratio, *MPT* maximum phonation time, *VHI-10* voice handicap index-10, *V-RQoL* voice-related quality of life, *GFI* glottal function index

**Fig. 1:**
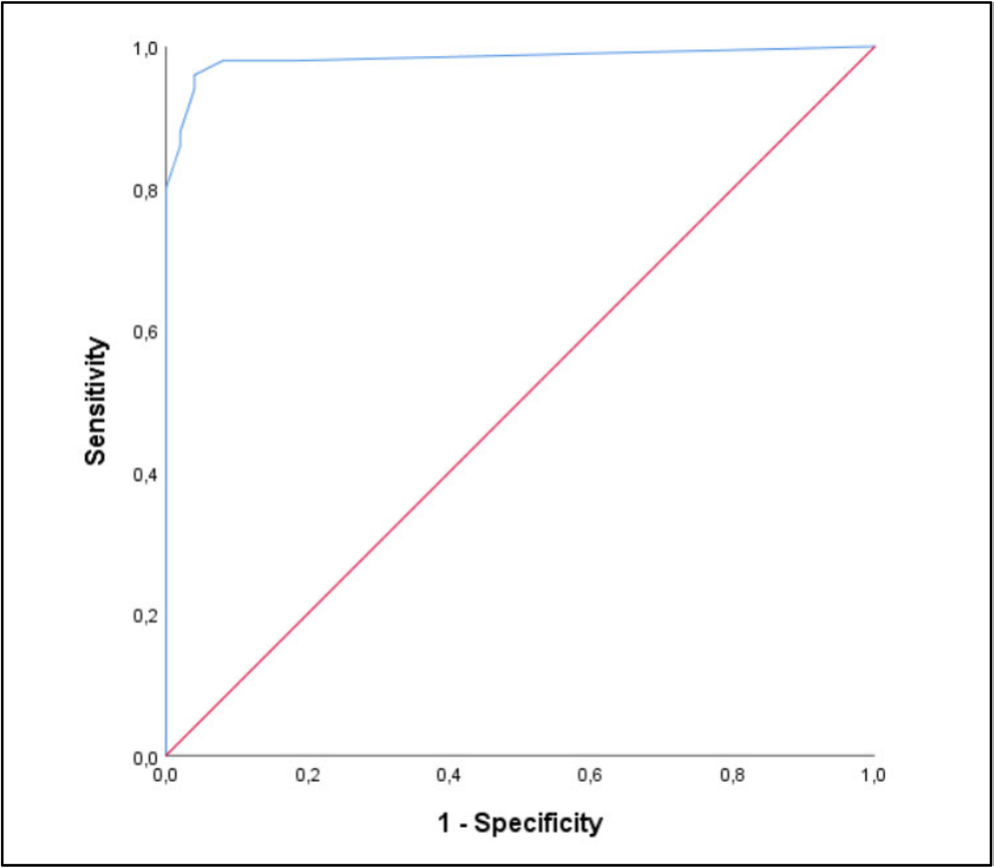
ROC curve generated for the diagnostic performance of GFI in patients with functional voice disorders

The highest Youden J value (0.920) was obtained for a cutoff GFI score of 3. For a cutoff GFI value of ≥ 3, the sensitivity was determined as 96% with an AUC value of 0.983. The ROC curve generated for the diagnostic performance of GFI is illustrated in Fig. 1. The sensitivity, specificity, and positive and negative predictive values for the cutoff GFI score of 3 are given in Table 2.

**Table 2:** Diagnostic accuracy of GFI using a cutoff score of 3

Variables	AUC (95% CI)	Sig. *p*	Cutoff	Sens.%	Spec.%	NPV%	PPV%
SG vs. CG							
GFI	0.983 (0.957–1.000)	< 0.001	≥ 3	96.0	96.0	96.0	96.0

*Abbreviations*: *SG* study group, *CG* control group, *AUC* area under the curve, *CI* confidence interval, *NPV* negative predictive value, *PPV* positive predictive value, *GFI* glottal function index

## Discussion

The present study investigated the diagnostic utility of the GFI in distinguishing individuals with functional dysphonia from normophonic controls and aimed to determine an optimal GFI cutoff score for screening purposes. The results demonstrated that the GFI provided excellent diagnostic accuracy, with an optimal cutoff score of ≥ 3 yielding a sensitivity of 96% and an AUC of 0.983. These findings indicate that the GFI can serve as a brief yet highly sensitive screening tool for identifying individuals with functional dysphonia.

Previous studies have primarily evaluated the GFI’s performance in heterogeneous populations comprising diverse dysphonia etiologies, particularly those associated with glottal insufficiency [31, 34–39]. Across these studies, the GFI consistently demonstrated good diagnostic performance, effectively distinguishing dysphonic patients from healthy individuals. The current study extends previous findings by showing that the GFI maintains strong discriminative power even when applied specifically to individuals with functional voice disorders. This suggests that the tool captures perceptual and symptomatic dimensions of glottal dysfunction that remain clinically relevant in functional dysphonia, independent of organic pathology.

Beyond its diagnostic performance, the present study also confirmed that individuals with functional dysphonia exhibit distinct impairments across multiple domains of vocal function, providing a comprehensive profile of their voice characteristics. Consistent with previous literature, participants with functional dysphonia exhibited significantly deteriorated acoustic [50–52], auditory-perceptual [52], aerodynamic [51–53], and self-reported [52, 54] measures compared to normophonic controls. Increased shimmer and decreased HNR values indicated greater cycle-to-cycle amplitude perturbations and reduced voice signal periodicity, reflecting glottal inefficiency and hyperfunctional phonatory behavior [11]. The significantly higher G score of GRBAS and shorter MPT observed in the SG further support the presence of phonatory strain and suboptimal aerodynamic efficiency, both hallmark features of functional dysphonia [53]. Moreover, self-reported outcomes, including increased VHI-10 and GFI scores and decreased V-RQoL scores, consistently demonstrated the subjective burden of the disorder on daily communication and quality of life [55].

In the present study, the difference in GFI scores between participants with functional dysphonia and healthy controls was highly significant (*p* < 0.001). The median GFI score of the SG was substantially higher than that of the CG [14 (9.75–19) vs. 0 (0–0)]. Reported mean GFI scores in previous studies involving heterogeneous dysphonia groups show a relatively consistent pattern, with values ranging from approximately 9 to 13 [31–34, 36–38]. The median GFI value observed in the present study is slightly higher than those reported in these heterogeneous dysphonia groups, suggesting that individuals with functional dysphonia may experience and report more pronounced voice-related symptoms despite the absence of structural pathology [52]. In the study by Buckmire et al. [56], a higher median GFI score (16) was reported compared to previous studies and the present study. This finding may be attributed to the inclusion of patients with conditions associated with more severe glottal insufficiency, such as vocal fold paresis, hypomobility, scarring, and atrophy.

The ROC analysis in the current study demonstrated that a GFI score of ≥ 3 was the most appropriate cutoff value for differentiating patients with functional dysphonia from healthy controls, achieving 96% sensitivity and 96% specificity. A similar cutoff value (≥ 3) was also reported by Pribuišienė et al. [34] for Lithuanian-speaking adults with various types of dysphonia. Separately, Cohen et al. [57] identified the same threshold in their validation study of the GFI for children—predominantly those with vocal fold nodules—whose vocal characteristics and patterns of voice use differ substantially from those of adults. The optimal GFI cutoff score determined in this study (≥ 3) is lower than the threshold of ≥ 4 reported by Bach et al. [31] and Ülger et al. [33] for differentiating normal from pathological voices. This discrepancy may reflect the distinct pathophysiological nature of functional dysphonia, in which symptoms are primarily perceptual and behavioral rather than structural. The lower cutoff value identified here may therefore enhance sensitivity in detecting functional impairments that are not evident through laryngeal examination. Clinically, adopting a GFI threshold of 3 could facilitate earlier identification and referral of individuals at risk for functional dysphonia, optimizing both intervention timing and clinical resource use.

Although the diagnostic performance of the GFI was comparable to that of other self-reported measures, the primary value of the GFI lies in its brevity and ease of administration. Rather than replacing comprehensive voice-related quality of life measures, the GFI may serve as a brief screening instrument that facilitates early identification of individuals who require further multidimensional voice assessment. In time-limited clinical settings, this practical advantage may enhance workflow efficiency while maintaining diagnostic sensitivity.

Several limitations should be acknowledged. First, the sample size, though sufficient for ROC analysis, was limited to a single clinical center, which may restrict the generalizability of the findings to broader populations. Second, professional voice users were excluded, and their distinct phonatory demands and perceptual thresholds may influence GFI responses differently. Third, due to the cross-sectional nature of our case-control study, test–retest reliability of the self-reported measures could not be assessed within the present sample. In addition, sex-based subgroup analyses were not performed, and potential sex-related differences in the evaluated metrics could not be explored. Furthermore, the lack of calibrated SPL measurements limits the interpretation of acoustic parameters that are sensitive to variations in vocal intensity.

## Conclusions

The present study demonstrates that the GFI is a highly sensitive self-report tool for identifying individuals with functional dysphonia. A cutoff score of ≥ 3 optimally differentiated patients from normophonic controls, yielding 96% sensitivity and 96% specificity. These results underscore the GFI’s excellent diagnostic performance and clinical utility as a brief and easily administered screening measure capable of detecting functional impairments even in the absence of structural or neurological pathology. Its simplicity, brevity, and strong discriminative power make the GFI a valuable complement to multidimensional voice assessment protocols, facilitating early identification, timely intervention, and efficient use of clinical resources. By enabling clinicians to quickly recognize and address functional voice disorders, the GFI has the potential to improve patient outcomes and enhance the overall effectiveness of voice management. Future research should involve larger and more balanced sample populations, incorporate professional voice users, and address potential sex-specific differences to further validate the clinical utility of the GFI. Additionally, longitudinal follow-up studies are warranted to examine the responsiveness of the GFI to therapeutic outcomes in the treatment of functional dysphonia.

## Data Availability

All data generated or analyzed during this study are included in this article. Further enquiries can be directed to the corresponding author.

## Abbreviation

ROC: Receiver operating characteristic
GFI: Glottal function index
